# Genetic divergence at species boundaries of the dolphinfish (*Coryphaena hippurus*) in the Tropical Eastern Pacific

**DOI:** 10.7717/peerj.14389

**Published:** 2022-11-17

**Authors:** Maried Ochoa-Zavala, Pindaro Diaz-Jaimes, Sofía Ortega-García, Felipe Galván-Magaña

**Affiliations:** 1 Unidad de Ecología y Biodiversidad Acuática, Instituto de Ciencias del Mar y Limnnología, Universidad Nacional Autónoma de México, CDMX, Mexico; 2 Escuela Nacional de Estudios Superiores, Unidad Morelia, Universidad Nacional Autónoma de México, Morelia, Michoacán, Mexico; 3 Instituto Politécnico Nacional, Centro Interdisciplinario de Ciencias Marinas, La Paz, Baja California Sur, México

**Keywords:** Genetic structure, Population expansion, Genetic drift, Bottleneck, Range boundaries

## Abstract

**Background:**

Marine species constitute commercially important resources, and knowledge about mechanisms that shape phylogeographic patterns and genetic structure provides valuable information for conservation. The dolphinfish, *Coryphaena hippurus*, is one of the most important species caught in the Tropical Eastern Pacific (TEP). However, the lack of consensus about the existence of genetically differentiated populations in the area has hindered the adoption of management strategies to ensure its viability.

**Methods:**

We assessed genetic variation and phylogeographic structure using two mitochondrial genes and 14 nuclear DNA microsatellite loci. Population genetic tools were used to characterize the spatial distribution of genetic variation of *C. hippurus* in the TEP, evaluate the extent of connectivity between dolphinfish populations, infer potential barriers to gene flow, and test for signals of contemporary and historical demographic expansions.

**Results:**

Mitochondrial DNA sequences showed genetic homogeneity across locations in the TEP, as well as a strong signal of population expansion dated to the late Pleistocene. In contrast, nuclear microsatellite markers resolved four genetically distinct groups with a remarked genetic differentiation between the most distant locations, at the northern and southern boundaries of the species’ range. High mean genetic diversity was found at all localities (*Hs* = 0.66–0.81). Notwithstanding, positive *F*_IS_ and low effective population size (*Ne* = 77.9–496.4) were also recorded.

**Conclusions:**

The distribution of genetic variation could be related to expansion-contraction cycles following seasonal temperature changes at transitional areas, promoting population subdivisions. However, we cannot rule out the effect of oceanographic dynamics to the observed patterns. Although this marine species remains highly abundant despite commercial exploitation, the low *Ne* values are of conservation concern and must be considered in fishery management plans.

## Introduction

Most commercial fisheries depend on wild populations whose productivity is a function of a balance between biodiversity conservation and ecosystem equilibrium (Bohnsack & Ault, 1996). In 2018, global fish production reached an estimated 179 million tons, more than 87% of which was used for human consumption, providing 20% of the average *per capita* animal protein intake (Food & Agriculture Organization of the United Nations, 2020). However, marine fishery resources have continued to decline, reducing the proportion of fish stocks that remain within biologically sustainable levels (Food & Agriculture Organization of the United Nations, 2020). For species that are exploited in their natural environment, inefficient management may result in overexploitation, leading to population extirpations or severe reductions of genetic diversity (Laikre, Palm & Ryman, 2005). Thus, knowledge of mechanisms shaping phylogeographic patterns and genetic structure provides valuable information to define management units and establish conservation priorities for exploited marine species (Laikre, Palm & Ryman, 2005; Rocha, Craig & Bowen, 2007; Pavičić et al., 2020).

Large pelagic species account for an important share of the total marine fish production (Food & Agriculture Organization of the United Nations, 2020). Historically, these species have been considered highly abundant (census size) and assumed not to suffer from population decline given that they reproduce and grow at fast rates (Ward, Woodwark & Skibinski, 1994). Furthermore, their large effective population size (*Ne*) counteracts the impact of inbreeding and stochastic genetic drift (Allendorf, 1986; Frankham, 1996). However, recent studies have shown population declines and pressure due to overfishing in five out of ten populations in five surveyed species (Kindong et al., 2020). Moreover, inbreeding signals have been found, suggesting the possibility that local extinction could occur in the near future (Hoaurau et al., 2005; O’Leary et al., 2013).

The traditional view of the marine realm as an ‘open ecosystem’ is inherent in many early genetic studies of cosmopolitan pelagic fishes, which typically exhibited a lack of genetic differentiation over wide geographic scales (Ward, Woodwark & Skibinski, 1994; Hauser & Carvalho, 2008). However, recent work has shifted this paradigm by revealing that an increasing number of widely distributed species with high dispersal capability have population genetic structure among regions separated by only tens to a few hundred kilometers. This demonstrates that population subdivision in marine organisms is sometimes more complex than previously conceived (Ruzzante, 1998; Benzie, 2000; Knutsen et al., 2003; Jørgensen et al., 2005; Olsen et al., 2008; Handal et al., 2020). Although such genetic differentiation tends to be low, it is often statistically significant and relevant for management planning (Waples, 1998; Knutsen et al., 2011).

The marine realm offers a mosaic of dynamic conditions that can promote genetic differentiation between populations occupying large areas due to the existence of mesoscale physical events such as upwelling systems, jets, gyres, tides, and oceanic fronts that can affect dispersal and act as temporary barriers to genetic exchange between relatively close populations (Cowen et al., 2000). Particularly for tropical pelagic fishes, water temperature represents the main factor limiting their distributional range. Seasonal range expansions and contractions may occur as fish track changing water temperatures at the edge of their range, promoting migration to suitable environments and contraction in suboptimal environments (Anderson et al., 2013). These expansion-contraction cycles affect populations’ present-day genetic composition and promote intra-specific genetic differentiation (Hewitt, 2000), especially at the boundaries of large geographic ranges.

*Coryphaena hippurus*, the dolphinfish or mahi-mahi, is a cosmopolitan, highly migratory pelagic fish found in tropical and subtropical waters around the world (Palko, Beardsley & Richards, 1982). It is a fast-growing fish that can reach two meters in fork length and weigh up to 30 kg at 3 years of age. The increase in abundance of dolphinfish populations during the summer has been interpreted as evidence of seasonal migratory behavior (Oxenford, 1999). Nevertheless, despite its dispersal capability, mark-recapture experiments have demonstrated that individuals remain resident near where they were initially tagged (Kingsford & Defries, 1999; Merten, Appeldoorn & Hammond, 2014; Perle et al., 2020). Both recreational and commercial fisheries target dolphinfish along the TEP coasts, where the species is locally abundant. It is one of the most important species caught in the TEP, with major reported catches in Ecuador and Peru since 1990. The Food and Agriculture Organization of the United Nations (FAO) reported that from 2015 to 2017, the global catch of dolphinfish decreased by 31%, from 125,000 t to 86,000 t. Peru has reported the highest catch levels and, together with Ecuador, contributed more than 50% of the world’s total catch from 2015 to 2017 (Food & Agriculture Organization of the United Nations, 2020). Despite this level of commercial exploitation, the dolphinfish is not currently considered an endangered or vulnerable species by the IUCN Red List of Threatened Species (Collette et al., 2011). Nevertheless, declines in the abundance of some dolphinfish populations in the TEP (Food & Agriculture Organization of the United Nations, 2020) have drawn attention to fishery managers highlighting the need to implement protective strategies.

Genetic studies of dolphinfish populations in the TEP have shown spatial and temporal homogeneity of mitochondrial DNA (mtDNA) sequences in the eastern Pacific (Díaz-Jaimes et al., 2006). However, haplotype frequency differences have also been detected between central Pacific (Hawaii) and eastern Pacific samples based in RFLPs of the NADH subunit 1 (ND1) locus (Rocha-Olivares et al., 2006). Tripp-Valdez et al. (2010) reported subtle spatial and temporal genetic differences between collections from the Gulf of California and surrounding areas using five microsatellite loci. However, as the evidence was inconclusive, the authors suggested that the dolphinfish is composed of a single panmictic population with high inter-annual variation and gene flow. More recently, analyses of mtDNA and five microsatellite loci of dolphinfish from Pacific populations off Colombia revealed two discrete genetic discontinuities (Téllez & Caballero, 2017). Since these discontinuities were associated with monthly regional changes in abundance, they were hypothesized to result from the migration of individuals from distant locations with different allele frequencies to Colombia coasts (Téllez & Caballero, 2017). The differences in the molecular markers used and the spatial and temporal scales of these studies have impeded a consensus on whether dolphinfish populations in the TEP are genetically structured. Thus, a robust assessment is still needed to provide insights on the species’ genetic characteristics in order to define effective management strategies that would ensure long-term viability.

In this study, we combined the analysis of two mitochondrial segments and 14 nuclear microsatellite loci to (1) characterize the spatial distribution of genetic variation of *C. hippurus* in the TEP, (2) evaluate the connectivity of the dolphinfish locations, (3) infer barriers to gene flow, and (4) estimate past and contemporary changes in effective population sizes to provide insight on possible associations with climate variability. The motivation of this study was to provide genetic information that is necessary to define the dolphinfish population connectivity with ample spatial sampling and more informative markers to gain knowledge on this highly exploited species.

## Materials and Methods

### Sample collection and DNA isolation

**Figure 1:**
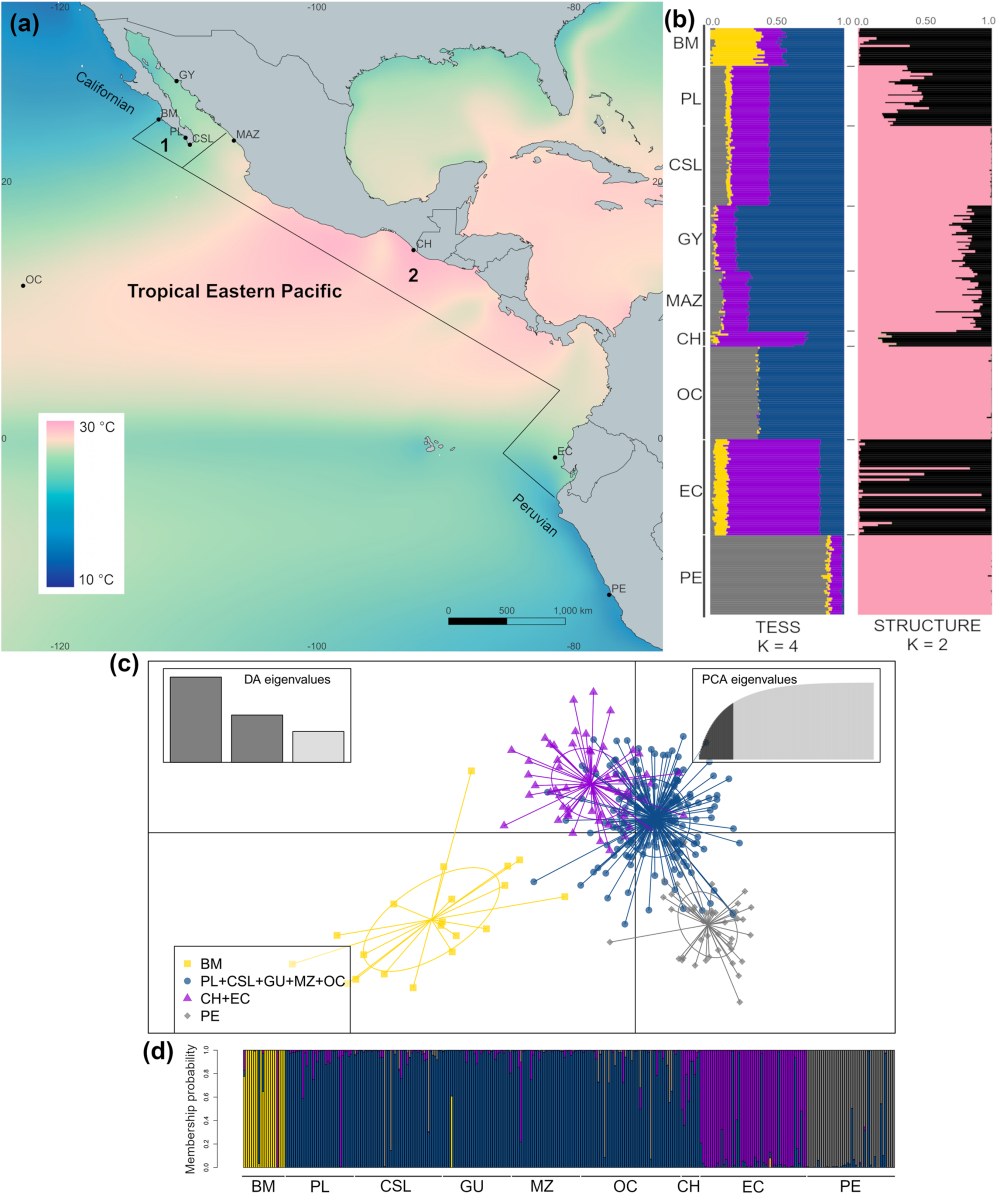
Sample collection sites and genetic structure of dolphinfish *Coryphaena hippurus* in the Tropical Eastern Pacific (TEP). (A) Sample sites and annual mean surface temperature 2002 to 2009 retrieved from Bio-Oracle (Tyberghein et al., 2012; Assis et al., 2017); black dots indicate sampling localities (names of localities in Table 1). Notice the northern and southern zones of temperature discontinuity on the edges of the TEP, where the Californian and Peruvian provinces begin. Numbers indicate the Cortez (1) and the Panamic (2) biogeographic provinces off the continental shores of the TEP. (B) The spatial pattern of genetic variation of *C. hippurus* derived from the microsatellite analysis. Bar plots of the clustering analysis implemented in TESS3 and STRUCTURE for *K* = 4 and *K* = 2, respectively. (C) Scatterplot from the discriminant analysis of principal components (DAPC; adegenet) using *K* = 4. (D) DAPC’s bar plot showing the probabilities of assignment of individuals to *K* = 4. Population names are in Table 1.

Muscle tissue samples from dolphinfish individuals were opportunistically collected from 2003 to 2006, from artisanal fishing boats operating in the TEP, including the Gulf of California (Table 1; Fig. 1). Samples collected in Mexico included the Pacific coast of Baja California Sur (Bahía Magdalena (BM), Punta Lobos (PL), and Cabo San Lucas (CSL)); within the Gulf of California (Guaymas (GY)); and in the eastern central Pacific (Mazatlán (MAZ), and Chiapas (CH)). In South America, sample locations comprised Ecuador (EC) and Peru (PE). We also included a sample from an oceanic area (OC) located at coordinates 11.886755 N, 122.880087 W (Fig. 1). Total genomic DNA was isolated using the proteinase K-lysis-buffer protocol (Laird et al., 1991) or the Wizard Genomic kit (Promega Cat. No. A1125); DNA was rehydrated in 50–100 μl of TE buffer.

**Table 1:** Population names, geographic coordinates, and estimates of genetic diversity for nuclear microsatellites and mitochondrial genes ND1 and CYTB from *Coryphaena hippurus* localities within the Tropical Eastern Pacific.

Name	Loc ID	Latitude	Longitude	Microsatellites			Mitochondrial ND1					Mitochondrial CYTB				
				N	Na	Pa	N	nh	p	h	π	N	nh	p	h	π
Bahía Magdalena	BM	24.579118	−111.998283	20	121	4	16	12	21	0.94 (0.05)	0.006 (0.003)	17	8	19	0.846 (0.066)	0.005 (0.003)
Punta Lobos	PL	23.413503	−110.234702	32	129	5	32	12	20	0.81 (0.05)	0.003 (0.002)	0	–	–	–	–
Cabo San Lucas	CSL	22.869653	−109.898667	42	135	3	36	18	23	0.90 (0.03)	0.003 (0.002)	36	16	21	0.871 (0.046)	0.004 (0.003)
Guaymas	GY	27.825287	−110.933325	35	134	2	51	33	41	0.94 (0.02)	0.004 (0.002)	33	14	18	0.865 (0.039)	0.004 (0.002)
Mazatlán	MAZ	23.19246	−106.471719	32	107	2	49	24	37	0.88 (0.04)	0.004 (0.002)	36	20	32	0.894 (0.042)	0.005 (0.003)
Chiapas	CH	14.664185	−92.482619	8	75	0	45	32	42	0.96 (0.02)	0.005 (0.003)	0	–	–	–	–
Oceanic sample	OC	11.886755	−122.880087	49	136	9	48	28	43	0.91 (0.03)	0.004 (0.002)	33	21	23	0.941 (0.025)	0.004 (0.002)
Ecuador	EC	−1.484832	−81.457839	51	150	11	48	29	47	0.92 (0.03)	0.004 (0.002)	34	15	16	0.863 (0.050)	0.004 (0.002)
Peru	PE	−12.18415	−77.250531	42	122	3	39	19	28	0.91 (0.03)	0.004 (0.002)	24	11	20	0.884 (0.040)	0.005 (0.003)

**Notes:**

N, sample size; Na, number of alleles; Pa, private alleles.

nh, number of haplotypes; p, number of polymorphic sites; h, haplotype diversity; π, nucleotide diversity.

### Microsatellite genotyping and quality control

Nineteen microsatellites were genotyped using primers for five loci (Chi002, Chi008, Chi008A, Chi023, and Chi037) designed by Robert Chapmann et al. 2005 (personal communication; GenBank Accession Numbers: AY135025–AY135028, and AY189832), 13 described by Bayona-Vásquez, Díaz-Jaimes & Uribe-Alcocer (2015), and one tetra-nucleotide locus (Chi284) derived from Next Generation Sequencing by Bayona-Vásquez, Díaz-Jaimes & Uribe-Alcocer (2015): with repetitive motive TTCC7: forward primer (5′ - 3′): TGTGGGAGAGGTCATTGCC; reverse primer (5′- 3′): AGAGGCAAGAGTGATGGTGC) (available at 10.5281/zenodo.6787993).

PCR amplification reactions were performed in 10 μl containing 10–100 ng of DNA, 1X PCR buffer (20 mM Tris-HCl, pH 8.4, 50 mM KCl), 0.2 mM of dNTPs, 0.2 μM of each primer, 2.5 mM of MgCl_2_, and 0.25 U of Taq DNA polymerase. All forward primers had an M13 tail incorporated (5′-GTAAAACGACGGCCAGT-3′), and a third M13 primer labeled with a fluorophore (6-FAM, VIC, NED or PET) was included in the reaction to bind to the forward primers. Forward, reverse, and florescent-M13 primers were used in 1:10:10 proportions, respectively, and were added to the reaction mix according to the protocol described by Boutin-Ganache et al. (2001). The PCR conditions consisted of 5 min at 95 °C for denaturation, followed by 25 cycles of 30 s at 94 °C, aligning at 56 °C for 45 s, and extension at 72 °C for 45 s; followed by eight additional cycles at 94 °C for 30 s, 50 °C for 45 s, 72 °C for 45 s, and a final extension step at 72 °C for 10 min. Reactions were multiplexed for fragment analysis in an ABI-Prism 3100® sequencer. Genotypes were assigned using GeneMapper® software (Cat. No. 4475073; Thermo Fisher Scientific, Waltham, MA, USA) using GeneScan™ 500 LIZ™ (Cat. No. 4322682; Thermo Fisher Scientific, Waltham, MA, USA) as a size standard.

The existence of null alleles in the microsatellite data was evaluated with MICROCHECKER 2.2.3 (Van Oosterhout et al., 2004) using 1 × 10^4^ randomizations and a confidence interval (CI) of 95%. Departures from Hardy-Weinberg equilibrium (HWE) for each locus were examined by the exact test method. We also tested for linkage disequilibrium (LD) between pairs of loci. For both tests we used GENEPOP 4.7.5 software (Raymond & Rousset, 1995; Rousset, 2008), implementing 1 × 10^4^ dememorizations and iterations with 500 batches. For HWE and LD we applied a Bonferroni correction with the standard procedure (Miller, 1980; Lessios, 1992) using an initial critical value of 0.05. The final data set was used to assess the genetic diversity and structure.

### Mitochondrial DNA sequencing

We amplified fragments of mitochondrial gene ND1 and cytochrome B (CYTB) using the primers reported by Díaz-Jaimes et al. (2006) and Hyde et al. (2005), respectively. PCR reactions for sequencing were carried out in 50 μl containing 10–100 ng of DNA, 1X amplification buffer, 10 mM TRIS-HCl (pH 8.4), 50 mM KCl, 1.5 mM MgCl_2_, 0.2 mM of each dNTP, 0.1 mM of each primer and 2.5 units of Platinum® *Taq* DNA polymerase (Cat. No. 10966018; Thermo Fisher Scientific, Waltham, MA, USA). PCR amplifications for ND1 consisted of 95 °C for 5 min followed by 35 cycles of 1 min at 95 °C for denaturation, 1 min at 58 °C for annealing, and a final extension at 65 °C for 3 min, with a final extension at 72 °C for 10 min. Amplicons were purified and sequenced on an ABI 3730xl® automated sequencer by Macrogen Inc. (Seoul, South Korea). The thermal cycling profile for CYTB consisted of an initial denaturation step at 95 °C for 2 min, followed by 35 cycles at 95 °C for 1 min, 52 °C for 1 min, and 65 °C for 1 min, with a final extension of 3 min at 65 °C. Mitochondrial sequences were edited and aligned using ClustalX 1.8 (Thompson et al., 1997). The complete ND1 data sets from Díaz-Jaimes et al. (2006) and Díaz-Jaimes et al. (2010) from the eastern Pacific were reanalyzed, and new sequences from Bahía Magdalena, Punta Lobos, and the Oceanic area were added. Sequences of CYTB reported in this study are available from GenBank (accession numbers: MZ725384–MZ725448). The two loci were analyzed independently because of inconsistency in the amplification of many individuals. Because of this, we could not concatenate the sequences from the two loci.

### Data analyses

#### Gene diversity

For nuclear microsatellites, genetic diversity was assessed in terms of total number of alleles (Na), private alleles (Pa), observed heterozygosity (*H*_*o*_), mean gene diversity (*Hs*), and allelic richness (*Ar*). Gene diversity and inbreeding coefficient (*F*_IS_) were estimated using the R 3.5.3 (R Core Team, 2019) statistics package ‘*hierfstat’* (Goudet & Jombart, 2015), and the lower and upper limits of CIs for *F*_IS_ were derived from 1 × 10^4^ bootstraps using the *boot.ppfis* function. For *Ar* the rarefaction method was applied to each sampled locality (*n* = 16 alleles) using the function *allelic.richness* using the same R package.

For both mitochondrial sequences, we identified the best substitution model using the hierarchical likelihood ratio test implemented in JMODELTEST 2.1.10 (Darriba et al., 2012) using the Akaike (AIC) and Bayesian (BIC) information criteria. The best-fitting model for ND1 was TIM1+G (Posada, 2003) with a gamma substitution parameter of 0.2160; for CYTB it was K80+G (Kimura, 1980) with a gamma substitution of 0.2770. The number of haplotypes (n_h_), number of polymorphic sites (p), and haplotype (h) and nucleotide (π) diversity were estimated using ARLEQUIN v. 3.5.2.2 (Excoffier & Lischer, 2010).

#### Genetic structure

An isolation by distance (IBD) model was evaluated from the microsatellite data using a Mantel test to estimate the correlation between genetic and geographic distances using the *‘ade4’* package with 1 × 10^4^ replicates. We used pairwise D_A_ genetic distances (Nei, Tajima & Tateno, 1983) and computed geographic distances between pairs of sampling locations, avoiding land masses using the *lc.dist* and *trans.mat* functions implemented in the ‘marmap’ package (Pante & Simon-Bouhet, 2013). The allelic differentiation and fixation index were estimated by pairwise Jost’s *D* (Jost, 2008) and G_ST_ (Nei, 1973), respectively, using the function *fastDivPart* implemented in the ‘diverRsity’ R package using 100 bootstraps (Keenan, 2017). We estimated *P*-values from CIs following Altman & Bland (2011). For mitochondrial sequences, genetic differentiation between pairs of samples (*φ*_ST_) and their respective *P*-values were calculated based on Tamura and Nei distance corrected by the gamma shape parameter; we used 1 × 10^4^ permutations and fixed a significant level of 0.05 in ARLEQUIN.

As marine populations often exhibit low genetic structure (Jørgensen et al., 2005; Olsen et al., 2008; Handal et al., 2020), and this factor can bias genetic admixture proportion of individuals (Durand et al., 2009), we implemented a spatially explicit approach with TESS3 in the tess3r package (Caye et al., 2016) for microsatellite data to improve the inference of population genetic structure (François & Durand, 2010). TESS3 combines a least-squares minimization algorithm and spatial statistical methods to estimate ancestry coefficients. In addition, as it makes no assumption about linkage or HWE, it is appropriate to use where there is inbreeding or geographically-restricted mating (Caye et al., 2016). We performed 20 independent runs for *K* values ranging from 1 to 10. For each *K* value, we retained only the repetitions with the lowest root mean squared error. The optimal number for *K* was determined using a cross-validation method, considering that the smaller mean values are better, and the best choice is when the curve exhibits a plateau or starts increasing (Caye et al., 2016). To provide additional support for the optimal number of population clusters present given the data, we performed a discriminant analysis of principal components (DAPC) using the R package ‘adegenet’ 2.1.3. The optimum number of clusters (*K*) was obtained using the function *find.clusters* and selecting the best-supported number from the lowest Bayesian Information Criteria (BIC) value. After cross-validation, 49 PCA eigenvalues were retained, containing 78% of the variance. In addition, we evaluated genetic structure using the Bayesian framework from STRUCTURE 2.3.4 (Pritchard, Stephens & Donnelly, 2000), which assigns individuals to population clusters based on multi-locus genotypes. We implemented the admixture model with correlated allele frequencies. To assist the clustering analysis, we used the LOCPRIOR model (Hubisz et al., 2009). We ran 20 replicates for each *K* value ranging from 1 to 5, considering a burn-in period of 5 × 10^5^ and 1 × 10^6^ Markov Monte Carlo Chains after burn-in. To summarize replicate runs and visualize the assignment probabilities for each individual to the *K* clusters, we used CLUMPAK (Kopelman et al., 2015) with the default settings. To determine the optimum value of *K*, we implemented the Δ*K* method (Evanno, Regnaut & Goudet, 2005) and examined the mean posterior probability (Ln P(D)) for each *K* to identify the highest posterior probability or a plateau (Pritchard, Wen & Falush, 2003). Both Δ*K* and Ln P(D) were obtained from STRUCTURE HARVESTER (Earl & vonHoldt, 2012).

To explore different hypotheses of population structure within the TEP based on microsatellites, we performed hierarchical analyses of molecular variance (AMOVA) grouping locations according to (a) the biogeographic provinces proposed for resident fishes (Robertson & Cramer, 2009), and (b) the oceanographic regions defined by dominant physical processes (*e.g*., upwellings and ocean fronts). The biogeographic provinces were the: Californian (BM), Cortez (PL, CSL, GY, and MAZ), Panamic (CH and EC), Peruvian (PE) and the ocean islands (OC). The oceanographic regions were: the upwelling from the west coast of Baja California (BM), the Cabo San Lucas front (PL and CSL), upwelling of the continental margin of the Gulf of California (GY and MAZ), the Tehuantepec upwelling (CH), Peruvian upwelling (PE); considering Ecuador (EC), and the Oceanic sample (OC) as two distinct groups. AMOVA analyses were implemented in ARLEQUIN using the number of different alleles; significance of *F*-statistics was determined by 1 × 10^4^ permutations.

The haplotype networks for the two mitochondrial sequences were obtained using PopART 1.7 (Leigh & Bryant, 2015), applying a minimum spanning network (Bandelt, Forster & Röhl, 1999) with 1 × 10^4^ iterations. Then, we implemented BAPS 5.4 (Corander et al., 2008) to cluster the sampled individuals into groups. We used a mixture analysis based on clustering with linked loci analysis to account for the linkage present between sites using 20 replicates for each *K* (from 1 to 5). The admixture analysis (Corander & Marttinen, 2006; Corander et al., 2008) was based on 100 iterations.

#### Barriers to gene flow and recent migration rates

Potential barriers to gene flow based on microsatellite data were assessed with Monmonier’s maximum-difference algorithm and Delaunay triangulation of spatial coordinates of locations implemented in BARRIER 2.2 (Manni, Guérard & Heyer, 2004). We allowed a maximum of three barriers based on the assumption that boundaries of biogeographic provinces for resident fishes (Robertson & Cramer, 2009) may influence gene flow. The statistical support for each barrier was obtained by 1 × 10^3^ replicates of *D*_*A*_ genetic distances calculated by resampling individuals within populations using the Microsatellite Analyzer software (Dieringer & Schlötterer, 2003).

Recent migration rates among sampling locations were estimated using microsatellite data in BAYESASS 3.0.4 (Wilson & Rannala, 2003). BAYESASS implements a Bayesian approach based on individual multilocus genotypes and a Markov chain Monte Carlo (MCMC) technique to estimate migration rates over the last two generations. We carried out four independent runs consisting of 2 × 10^8^ iterations, discarding 5 × 10^6^ as burn-in and using a thinning interval of 1 × 10^3^ iterations. The mixing parameters for allele frequency, inbreeding coefficient, and migration rate were *a* = 0.6, *f* = 0.8, and *m* = 0.3, respectively. We assessed parameter convergence by examining trace files with TRACER 1.7.1 (Rambaut et al., 2018). We calculated the Bayesian deviance using the *calculateDeviance*.R script from Meirmans (2014) to measure the model fit for each independent run. We selected the run with the lowest deviance for our final estimates of the migration rates (Meirmans, 2014).

#### Effective population size

The contemporary effective population size (*Ne*) is a critical parameter when evaluating population conservation status for management planning. We estimated contemporary *Ne* for each population group obtained by TESS3 from microsatellite data (single population sample approach) using the bias-corrected version of the LD method (Waples & Do, 2008), as implemented in NeESTIMATOR 2.1 (Do et al., 2014). We launched NeESTIMATOR using an allele frequency of 0.02, as recommended for microsatellite markers (Do et al., 2014). The confidence intervals were obtained from parametric CI.

#### Demographic history

To detect recent reductions in effective population size over a relatively short period (several *Ne* generations) based on microsatellite data, we implemented BOTTLENECK 1.2.02 (Cornuet & Luikart, 1996; Piry, Luikart & Cornuet, 1999) with 2 × 10^4^ iterations. We performed a one-tailed Wilcoxon rank test to assess the probability of heterozygosity excess in each population group identified by TESS3 (with a sample size over 20 individuals) under three mutation models: the infinite allele model (IAM), two-phase mutation (TPM) with a variance of the geometric distribution = 0.36 as suggested for microsatellites by the authors, and the stepwise mutation model (SMM).

The historical demography of dolphinfish was inferred from several approaches, considering the whole sample of mtDNA sequences as a single population. First, mismatch distribution analysis (Rogers & Harpending, 1992) was conducted using ARLEQUIN with 1 × 10^4^ bootstrap replicates. Mismatch distribution compares observed frequencies of pairwise differences with a theoretical distribution under the sudden population expansion model. Harpending’s raggedness index (H_ri_) and the Sum of Squared deviations (SSD) were used to determine the fit to the expected curve under sudden population expansion. Because the CYTB fragment failed to reach convergence in ARLEQUIN, we obtained mismatch distributions in DnaSP 6.0.7 (Librado & Rozas, 2009). We expected a unimodal distribution if the population has undergone a sudden expansion. Second, we calculated Tajima’s *D* (Tajima, 1989) and Fu’s *F*s (Fu, 1997) neutrality test as implemented in ARLEQUIN. Third, trends in *Ne* were assessed using a Bayesian Skyline Plot (Drummond et al., 2005) implemented in BEAST 2.6.2 (Bouckaert et al., 2019). For this analysis, we used a strict molecular clock with mutation rates of 0.0076 (Díaz-Jaimes et al., 2010) and 0.0130 substitutions/site/million years (Martin & Palumbi, 1993) for ND1 and CYTB, respectively. The coalescent Bayesian skyline was chosen as the prior tree, assuming four group intervals with the substitution model TIM1+G for ND1 and K80+G for CYTB. Two independent replicates were carried out with 3 × 10^8^ MCMC and a pre-burning of 1 × 10^6^, storing every 5 × 10^3^ steps. Files from the two replicates were inspected using TRACER to assess convergence and determine whether effective sample size (ESS) exceeded 200 for each parameter. We plotted the BSP with their associated CIs (upper and lower highest posterior density at 95%) using TRACER.

## Results

Three of the 19 genotyped microsatellite loci (Chi062, Chi797 and Chi878) were missing in all individuals from one or three sampled localities (Table S1). Similarly, locus Chi357 showed more than 30% missing data across sampled localities. Microchecker showed null allele frequencies between 0.049 and 0.321. Six loci had null allele frequencies higher than 20% in Bahía Magdalena (BM) or Guaymas (GY) (Table S1). Evidence of null alleles for locus Chi024 showed a systematic bias across most localities. After Bonferroni correction, 12 loci showed deviations from HWE in a total of 23 combinations resulting from heterozygote deficit; locus Chi024 showed a systematic bias across sampling localities (Table S2). No significant linkage disequilibrium was detected in any pairs of loci after Bonferroni correction (Table S3). In summary, we excluded a total of five microsatellite loci due to null alleles in most localities (Chi024) and unacceptably high proportions of missing data (Chi357, Chi062, Chi797, and Chi878). With the remaining 14 loci, we estimated Jost’s *D* and G_ST_ values with and without the three loci that exhibited <25% of null allele frequencies (Table S1). As we found that null allele frequencies of <25% generate an insignificant effect on population structure estimates, for all subsequent analyses we used information from all 14 loci. Genotypes from the 14 nuclear microsatellites of *C. hippurus* are available at Zenodo with DOI 10.5281/zenodo.6949509.

### Gene diversity

**Figure 2:**
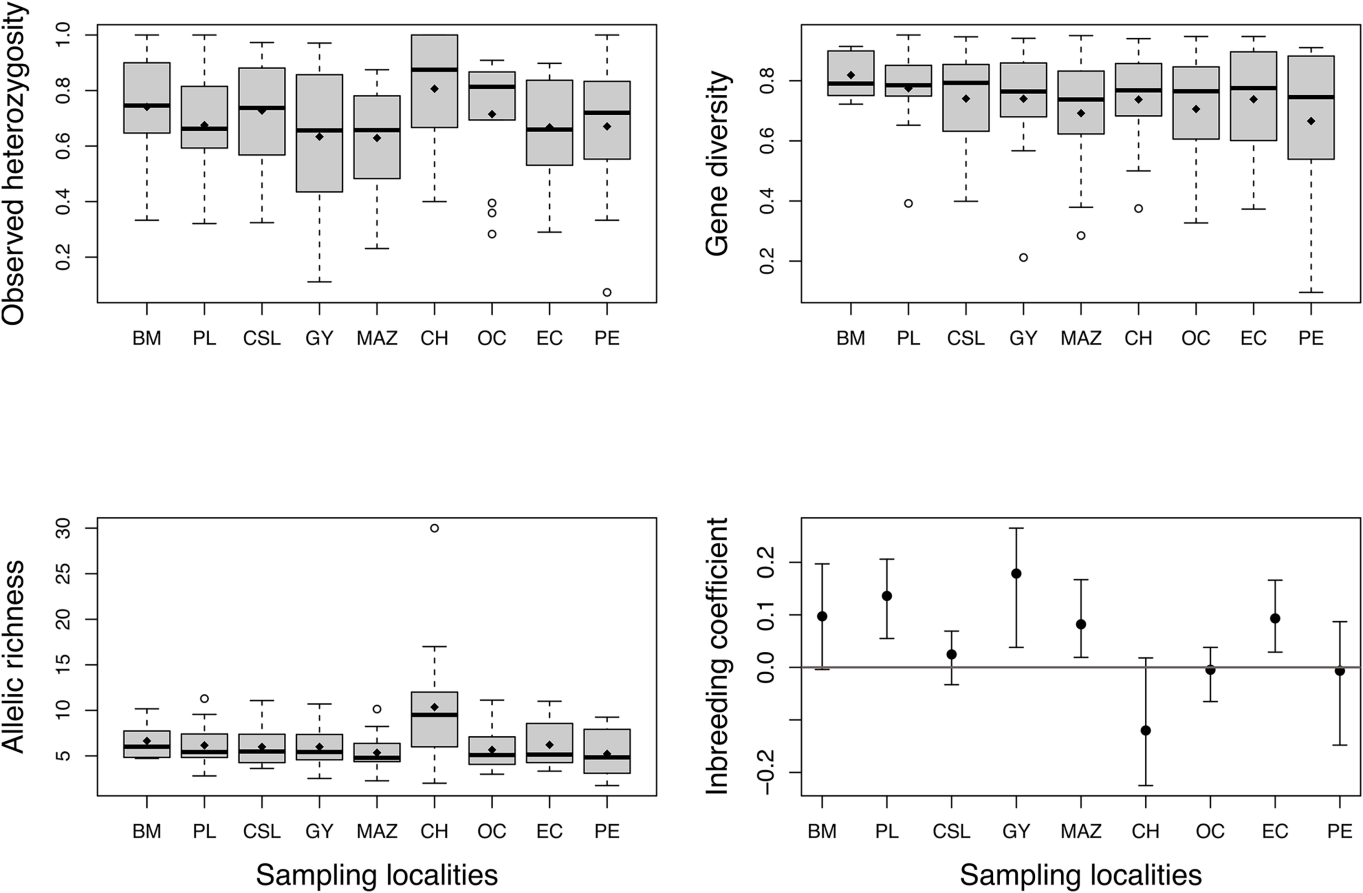
Estimates of genetic diversity and inbreeding coefficient from nine sampled localities of *Coryphaena hippurus*. Diamonds indicate the mean value for each sampling site. The inbreeding coefficient shows 95% confidence intervals of the mean for each locality. Names of localities in Table 1.

High levels of genetic diversity were observed in microsatellite loci across all studied locations. We obtained 218 alleles with a mean of 15.57 ± 6.3 alleles per locus. The total number of alleles (Na) ranged from 75 to 150 (Table 1), and all localities except Chiapas (CH) presented private alleles (Table 1). Chiapas (CH) exhibited the highest mean observed heterozygosity (*H*_*o*_), which over all localities ranged from 0.64 to 0.80 (Fig. 2). The mean gene diversity (*Hs*) across localities ranged from 0.66 to 0.81. Mean allelic richness (*Ar*) ranged from 5.2 to 10.3, averaging 6.4. Although Chiapas (CH) had the highest *H*_*o*_ and *Ar* values, *H*_*o*_, *Hs*, and *Ar* exhibited wide variability among sampling locations (Fig. 2); therefore, it was unclear whether Chiapas was the most diverse location. Most sites showed positive *F*_IS_ values, however, four sites (PL, GY, MAZ, and EC) exhibited a range of plausible values that suggested some degree of heterozygote deficiency, especially for Guaymas (GY) (Fig. 2).

For the mitochondrial analyses, we used the 750 base pair (bp) mtDNA-ND1 sequences from 364 individuals from the Díaz-Jaimes et al. (2006 and 2010) study and added 96 new individuals from the TEP to this dataset, resulting in a total of 117 haplotypes and 106 polymorphic loci. We also obtained a 506 pb fragment from the CYTB locus from 213 individuals, resulting in a total of 65 haplotypes and 72 polymorphic loci. Haplotype (*h*) diversity was higher than nucleotide diversity (π) for both fragments; *h* ranged from 0.81 (PL) to 0.96 (CH) for ND1 and from 0.85 to 0.94 for CYTB; π ranged from 0.003 (PL) to 0.005 (CH) for ND1 and from 0.004 to 0.005 for CYTB. Bahía Magdalena (BM) exhibited the lowest number of haplotypes for both fragments (n_h_ = 12 and 8), whereas Guaymas (n_h_ = 33) and the Oceanic sample (n_h_ = 21) had the highest estimations for ND1 and CYTB, respectively (Table 1). The 65 haplotypes for mtDNA-CYTB are available in GenBank with accession numbers: MZ725384–MZ725448.

### Genetic structure

For microsatellite data, we were unable to detect a significant correlation between geographic and genetic distances (*r* = −0.16, *P* = 0.66). The mean Jost’s *D* between localities (0.06, *P* = 0.018) suggested low allelic differentiation, whereas values of *G*_ST_ (0.03, *P* = 0.0) indicated very low and significant differences in allele frequencies across sample sites. Nonetheless, comparisons involving Bahía Magdalena (BM), Peru (PE), and Ecuador (EC) (Table 2) exhibited the highest levels of allelic differentiation. In contrast, none of the mitochondrial pairwise *φ*_ST_ results were significant after Bonferroni corrections, with values ranging from 0 to 0.037 for ND1 and 0 to 0.014 for CYTB.

**Table 2:** Estimates of genetic differentiation. Allelic differentiation (Jost’s *D*) and fixation index (*GST*) estimates for pairwise comparisons of *Coryphaena hippurus* populations in the Tropical Eastern Pacific.

	BM	PL	CSL	GY	MAZ	CH	OC	EC	PE
(a) Allelic differentiation (Jost’s *D*)									
BM	0	**0.001**	**0.000**	**0.000**	**0.000**	**0.017**	**0.000**	**0.014**	**0.000**
PL	0.1194	0	0.122	0.853	0.114	0.741	0.232	0.204	**0.002**
CSL	0.1877	0.0309	0	0.294	**0.038**	0.089	0.116	**0.041**	**0.001**
GY	0.1556	0.0042	0.0192	0	0.126	0.388	0.126	**0.031**	**0.009**
MAZ	0.1624	0.0316	0.0356	0.0326	0	0.655	**0.037**	**0.001**	**0.018**
CH	0.1448	0.0114	0.0563	0.0312	0.0188	0	0.204	**0.040**	**0.001**
OC	0.1389	0.0184	0.0327	0.0225	0.0187	0.0582	0	**0.000**	**0.023**
EC	0.0814	0.0233	0.0335	0.0376	0.0583	0.068	0.0473	0	**0.001**
PE	0.2083	0.0779	0.0489	0.0556	0.0353	0.1048	0.0291	0.0676	0
(b) Fixation index (*G*_ST_)									
	BM	PL	CSL	GY	MAZ	CH	OC	EC	PE
BM	0	**0.000**	**0.000**	**0.000**	**0.000**	**0.000**	**0.000**	**0.000**	**0.000**
PL	0.0481	0	**0.000**	**0.003**	**0.002**	0.194	**0.000**	**0.000**	**0.000**
CSL	0.0575	0.0225	0	**0.000**	**0.000**	**0.000**	**0.002**	**0.000**	**0.000**
GY	0.0589	0.0158	0.0137	0	**0.002**	**0.001**	**0.000**	**0.000**	**0.000**
MAZ	0.0643	0.0171	0.0203	0.0168	0	**0.006**	**0.000**	**0.000**	**0.000**
CH	0.051	0.0124	0.0358	0.0325	0.0323	0	**0.000**	**0.001**	**0.000**
OC	0.0678	0.0256	0.0129	0.0212	0.0162	0.0507	0	**0.000**	**0.000**
EC	0.0485	0.021	0.0308	0.0363	0.0345	0.0327	0.0392	0	**0.000**
PE	0.0809	0.0452	0.0163	0.0366	0.028	0.0545	0.0163	0.0488	0

**Note:**

*P* values above the diagonal; in bold when *P* < 0.05.

The cross-validation criterion from TESS3 for microsatellite data suggested an optimal *K* value of 6–7. However, as the variance notably increased for *K* > 4 (Fig. S1A) and cross-validation scores decrease noticeably with *K* = 4, we considered *K* = 4 to be the best value to describe the genetic structure pattern of *C. hippurus* (Fig. 1B). Nonetheless, both *K* = 2 and *K* = 3 were also used (Fig. S1B*)*. The spatial pattern of genetic variation obtained by TESS3 suggested that Bahía Magdalena (BM) was an isolated population, with a second well-defined cluster consisting of Punta Lobos (PL), Cabo San Lucas (CSL), Guaymas (GY), Mazatlán (MAZ), and the Oceanic samples (OC) (Fig. 1B). Further south, TESS3 grouped Chiapas (CH) with Ecuador (EC), and Peru (PE) as a separate distinct population (Fig. 1B). These same four well-defined genetic clusters were corroborated by the DAPC analysis (Fig. S1C, Figs. 1C and 1D). The DAPC results showed almost no overlap and supported Bahía Magdalena (BM) and Peru (PE) as distinct populations despite, their geographic proximity to other sampled locations (Figs. 1C and 1D).

The Ln P(D) values from the STRUCTURE analysis increased when increasing *K* from *K* = 1 to *K* = 2, and reached a plateau with *K* = 3 (Fig. S1D). The Δ*K* method suggested *K* = 2 as the highest level of genetic partitioning in *C. hippurus* samples (Fig. S1D). Because Ln P(D) values differed slightly between *K* = 2 and *K* = 3, we chose *K* = 2 as the optimal number of clusters given the genetic data. The population structure inferred by the Bayesian algorithm using these values for *K*, assigned individuals to one of two clusters with high probability values (Fig. 1B). Individuals with high probability of membership to the first cluster (black bars) included samples from Bahía Magdalena (BM), Punta Lobos (PL), Chiapas (CH), and Ecuador (EC). The second cluster (pink bars) contained Cabo San Lucas (CSL), Guaymas (GY), Mazatlán (MAZ), the Oceanic sample (OC), and Peru (PE). Although the three analyses inferred slightly different population structures, all three consistently reflected a genetic divergence at the TEP’s northern and southern transitional areas, which correspond with the boundaries of the species’ distribution range (Fig. 1).

The hierarchical AMOVA grouping by biogeographic provinces using the microsatellite results revealed that ~93% of the genetic variation was explained by differences within populations (Table 3). However, this analysis also indicated a low but significant structure among biogeographic provinces (*F*_CT_ = 0.03; *P* = 0.004). This contrasts with the lack of significant differences of groupings based on dominant oceanographic processes and coastal regions (see Methods) (*F*_CT_ = 0.02; *P* = 0.11). It is noteworthy that the clustering results from TESS3 coincided fairly well with the biogeographic provinces proposed for the TEP, suggesting the influence of differences in environmental conditions among provinces in shaping the genetic structure.

**Table 3:** AMOVA analyses. Analysis of molecular variance (AMOVA) grouping locations by (a) four Tropical Eastern Pacific provinces, and (b) upwelling zones and ocean fronts.

Source of variation	Sum of squares	Variance component	Percentage of variation	*F*-statistic	*P*-value
(a) TEP provinces					
Among groups	168.66	0.15	2.64	*F*_CT_ = 0.03	0.003
Among populations within groups	84.10	0.26	4.61	*F*_ST_ = 0.07	0
Within populations	3255.60	5.31	92.75	*F*_SC_ = 0.05	0
(b) Upwellings and Ocean fronts					
Among groups	200.73	0.09	1.68	*F*_CT_ = 0.02	0.11
Among populations within groups	52.03	0.30	5.20	*F*_ST_ = 0.07	0.0
Within populations	3255.59	5.31	93.11	*F*_SC_ = 0.05	0.0

**Note:**

**TEP provinces:** G1: Californian (BM); G2: Cortez (PL, CSL, GY, and MAZ); G3: Panamic (CH and EC); G4: Peruvian (PE); and G5: the ocean islands (OC). **Upwellings and Ocean fronts:** G1: Upwelling from the west coast of Baja California (BM); G2: Cabo San Lucas front (PL and CSL); G3: upwelling of the continental margin from the Gulf of California (GY and MAZ); G4: Tehuantepec upwelling (CH); G5: Peruvian upwelling (PE); considering Ecuador (EC) and the Oceanic sample (OC) as two distinct groups (G6 and G7, respectively). Names of localities in Table 1.

**Figure 3:**
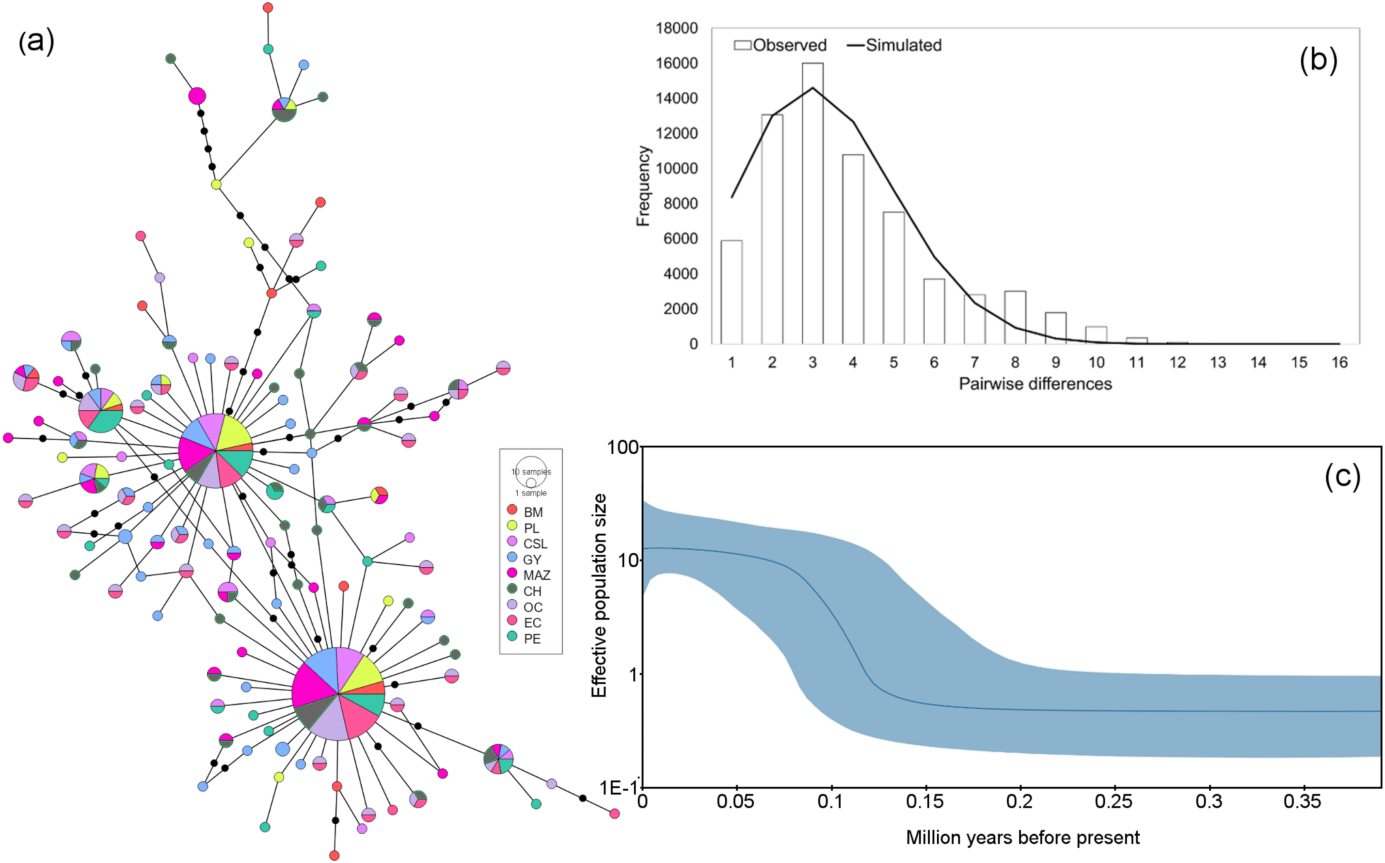
Haplotype network and demographic history of *Coryphaena hippurus* based on the mitochondrial gene ND1. (A) The minimum spanning network. The circle sizes are proportional to the haplotype frequencies. Black dots represent missing haplotypes. Results using the entire dataset for (B) mismatch distribution under a sudden expansion model. (C) Bayesian Skyline plot showing changes in effective population size (Ne) over time using the entire dataset. The solid line represents the mean estimated population size with 95% HPD intervals (blue shaded area).

**Figure 4:**
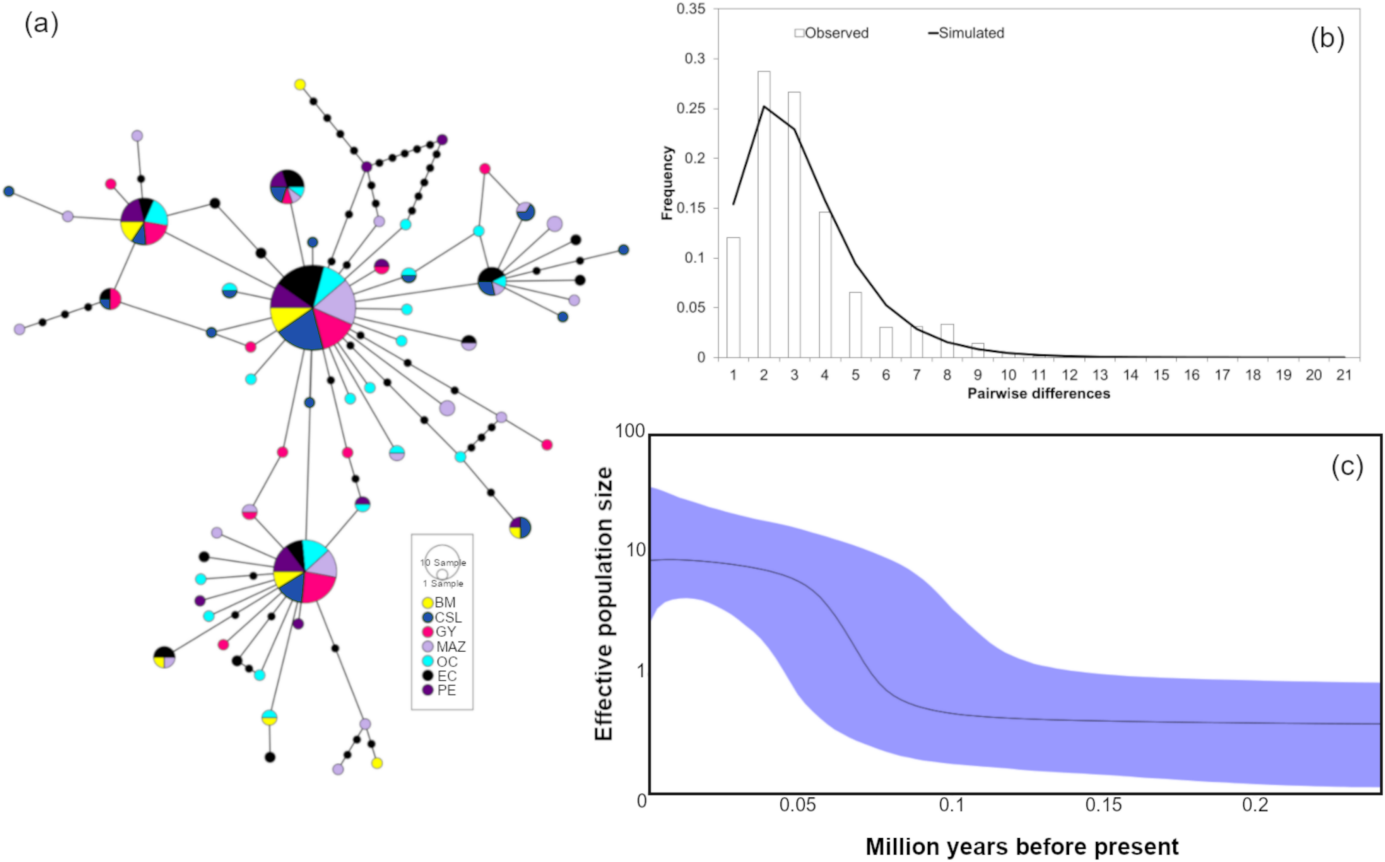
Haplotype network and demographic history of *Coryphaena hippurus* based on mitochondrial gene CYTB. (A) The minimum spanning network. The circle sizes are proportional to the haplotype frequencies. Black dots represent missing haplotypes. Results using the entire dataset for (B) mismatch distribution under a sudden expansion model. (C) Bayesian Skyline plot showing changes in effective population size (Ne) over time using the entire dataset. The solid line represents the mean estimated population size with 95% HPD intervals (purple shaded area).

Analyses of the genetic relationships between mitochondrial haplotypes from the two loci revealed two main phylogroups and no evidence of correlations between the haplotype differences within the network and different frequencies among the sampled populations (Figs. 3A and 4A). In both haplotype networks, two dominant haplotypes were found, with similar abundances for each at all locations. Likewise, the Bayesian assignment analyses for the two mitochondrial loci suggested that the most probable number of clusters (*K*) given the data was *K* = 2. However, no geographic structuring was detected for either of the mitochondrial fragments (Fig. S2).

### Barriers to gene flow and recent migration rates

Using microsatellite data, BARRIER analysis identified genetic boundaries with good statistical support (>65%; Fig. S3), which coincided with the population groups resolved by TESS3 and DAPC. Interestingly, BARRIER also identified a sharp genetic discontinuity surrounding Bahía Magdalena (BM) and Peru (PE). Nonetheless, contrary to the other analyses, BARRIER further subdivided Chiapas (CH) and Ecuador (EC) as two isolated populations. Migration rates from BAYESASS largely agreed with the barriers to gene flow. The migration patterns were asymmetric, with overall low estimates of migration rate (≤0.01), which ranged from 0.2765 to 0.0056 (Table 4). We detected that populations from Guaymas (GY) and the Oceanic sample (OC) exhibited the highest values and thus may connect numerous populations in southern regions of the TEP (*e.g*., GY to PL, MAZ, and CH; and OC to CSL and PE, respectively).

**Table 4:** Migration rates estimated between source and recipient populations of *Coryphaena hippurus* in the Tropical Eastern Pacific.

		Source								
		BM	PL	CSL	GY	MAZ	CH	OC	EC	PE
**Recipient**	BM	*0.8784 (0.0324)*	0.0117 (0.0113)	0.0128 (0.0129)	0.0180 (0.0159)	0.0117 (0.0113)	0.0117 (0.0113)	0.0243 (0.0178)	0.0198 (0.0170)	0.0116 (0.0113)
	PL	0.0081 (0.0079)	*0.6748 (0.0079)*	0.0081 (0.0080)	**0.2637 (0.0216)**	0.0081 (0.0079)	0.0081 (0.0079)	0.0113 (0.0104)	0.0096 (0.0092)	0.0081 (0.0079)
	CSL	0.0066 (0.0064)	0.0065 (0.0064)	*0.6781 (0.0217)*	0.0083 (0.0083)	0.0065 (0.0064)	0.0065 (0.0064)	**0.2743 (0.0283)**	0.0067 (0.0065)	0.0065 (0.0064)
	GY	0.0086 (0.0083)	0.0077 (0.0075)	0.0086 (0.0091)	*0.8842 (0.0298)*	0.0078 (0.0076)	0.0078 (0.0076)	0.0584 (0.0264)	0.0092 (0.0088)	0.0077 (0.0075)
	MAZ	0.0081 (0.0079)	0.0081 (0.0079)	0.0084 (0.0082)	**0.2428 (0.0271)**	*0.6748 (0.0079)*	0.0081 (0.0079)	0.0310 (0.0203)	0.0105 (0.0097)	0.0081 (0.0080)
	CH	0.0196 (0.0185)	0.0196 (0.0185)	0.0197 (0.0185)	**0.1763 (0.0393)**	0.0196 (0.0185)	*0.6862 (0.0184)*	0.0196 (0.0185)	0.0197 (0.0185)	0.0196 (0.0185)
	OC	0.0070 (0.0067)	0.0057 (0.0056)	0.0069 (0.0078)	0.0217 (0.0161)	0.0057 (0.0056)	0.0057 (0.0057)	*0.9345 (0.0214)*	0.0069 (0.0067)	0.0058 (0.0056)
	EC	0.0380 (0.0201)	0.0056 (0.0055)	0.0073 (0.0093)	0.0101 (0.0094)	0.0056 (0.0055)	0.0056 (0.0055)	0.0333 (0.0153)	*0.8890 (0.0261)*	0.0056 (0.0055)
	PE	0.0073 (0.0071)	0.0065 (0.0064)	0.0066 (0.0064)	0.0103 (0.0087)	0.0066 (0.0064)	0.0065 (0.0064)	**0.2765 (0.0176)**	0.0065 (0.0064)	*0.6732 (0.0064)*

**Note:**

Migration rates higher than 0.17 are shown in bold; intra-locality values (diagonal) in italics indicate the proportion of individuals that are not migrants, standard deviations in parentheses. Names of localities as in Table 1.

### Effective population size

Estimates of contemporary *Ne* suggested, in general, low values of *Ne* for *C. hippurus* within the TEP. Estimates ranged from 77.9 to 496.4 (Table 5). The genetic groups composed of Chiapas and Ecuador (genetic group C in Table 5) and the one corresponding to Peru (genetic group D) had the lowest values of *Ne*.

**Table 5:** Effective population sizes and heterozygosity excess.

Genetic cluster	Location IDs	Sample size	Effective population size			Wilcoxon rank test		
			*Ne*	Lower CI	Upper CI	IAM	TPM	SMM
A	BM	20	496.4	78.5	Infinite	**0.045288**	0.067627	0.178772
B	PL, CSL, GY, MAZ, and OC	190	368.6	281	522.9	**0.000061**	**0.002136**	0.932373
C	CH and EC	59	293	158.5	1427.5	**0.00058**	0.067627	0.6651
D	PE	42	77.9	55.4	123.6	**0.000427**	**0.024719**	0.076538

**Notes:**

Estimates of effective population size (*Ne*) for sites identified in the four genetic clusters (A–D); and the Wilcoxon range test to assess the probability of heterozygosity excess in each population cluster. Names of localities as in Table 1.

Lower CI and Upper CI are the lower and upper limits of the estimated 95% confidence intervals. Infinite allele model (IAM); two phase mutation model (TPM); stepwise mutation model (SMM). *P* values < 0.05 in bold.

### Demographic history

Both mitochondrial and nuclear DNA genetic markers provide evidence of ancient and recent changes in effective population size. For instance, based on the IAM and TPM mutation models, Wilcoxon rank tests suggested a recent contraction in population size for two genetic groups (Table 5). The first one largely corresponded to localities from Mexico (genetic group B in Table 5); and the second included Peru (genetic group D).

The mitochondrial sequences showed a strong signal of population expansion. The distribution of mismatches for the whole dolphinfish population in the eastern Pacific for both fragments was clearly unimodal (Figs. 3B and 4B). For mtDNA-ND1, Harpending’s raggedness index (Hri) and Sum of Squared deviations (SSD) had low and non-significant values (Hri = 0.026, *P* = 0.057; SSD = 0.004, *p* = 0.362), supporting the occurrence of a sudden demographic expansion and past fluctuations in population size. Tajima’s *D* and Fu’s *F*s both suggested departures from neutrality for ND1 (Tajima’s *D* = −2.44, *P* = 0.0; *F*s = −25.96, *P* = 0.0, respectively) and CYTB (Tajima’s *D* = −2.46, *P* = 0.0; *F*s = −26.85, *P* = 0.0, respectively). Demographic Bayesian reconstructions from the mtDNA-ND1 gene suggested a growth of the dolphinfish population that started approximately 126,000 years before present (Fig. 3C), with the population size scaled by a generational time increasing by approximately threefold from 0.73 (CI [10.20–0.27]) to 12.63 (CI [33.85–4.69]). Consistent with this, CYTB suggested a population growth approximately 92,838 years before present, with an increase in population size from 0.50 (CI [5.41–0.18]) to 8.88 (CI [36.62–2.68]) (Fig. 4C).

## Discussion

Knowledge of stock composition and population dynamics of exploited species is highly relevant for sustainable fisheries management (Ovenden et al., 2015). In the case of the dolphinfish populations in the Eastern Pacific, the molecular markers used until now have been incapable of identifying genetic stocks due to insufficient resolution to distinguish genetically differentiated populations. Maternal (*i.e*., mitochondrial) or biparentally inherited (*i.e*., nuclear microsatellites) molecular markers has been used separately, but had never been combined. Likewise, the sampling at different geographical scales has provided inconsistent patterns of genetic differences. This is the first study to apply mitochondrial DNA and nuclear microsatellite markers and a sampling of almost the entire latitudinal range of *C. hippurus* in the Tropical Eastern Pacific to infer historical and contemporary factors determining the genetic variation of dolphinfish populations. Based on nuclear microsatellite markers, it was possible to identify four significantly divergent genetic groups within the TEP. Although genetic population subdivisions were low, different lines of evidence confirm the observed differentiation pattern. In addition, the analysis of the mtDNA data strongly suggests that the population has undergone expansion.

### Genetic differentiation of dolphinfish in the TEP

Without apparent barriers to dispersal for marine species having a pelagic larval stage, we would expect genetically homogeneous populations. However, the low differentiation that has been reported does not necessarily imply high levels of gene flow among populations (Whitlock & Mccauley, 1998), even for species with high dispersal capabilities or those with an extended larval stage (Handal et al., 2020).

An interesting result from this study is the remarkable genetic differentiation between the two most distant locations (Bahía Magdalena (BM) and Peru (PE), Fig. 1) at the northern and southern extremes of the species range boundaries, which separate the tropical and subtropical waters in the TEP. These transitional areas are associated with seasonally complex oceanographic features, such as a convergence of different water masses and strong gradients with seasonal variation in physical (*e.g*., temperature and salinity) and chemical properties (*e.g*., dissolved oxygen, and nutrients) (Lluch-Cota et al., 2019). Considering that dolphinfish distribution is directly dependent on sea surface temperature (Zúñiga-Flores, Ortega-García & Klett-Traulsen, 2008), it is possible that transitional areas such as these play a significant role in originating cycles of range expansions-contractions in populations inhabiting them and thus, resulting in genetic differentiation. There is evidence of dolphinfish range expansion during warm ENSO phases, when an abnormal increase of sea surface temperature causes a poleward extension to these fresh and warm water areas usually characterized by relatively cooler temperatures. Studies on the spatio-temporal distribution of dolphinfish within the TEP have suggested that this species expands its habitat in response to increased ocean temperature (Norton, 1999; Torrejón-Magallanes, Grados & Lau-Medrano, 2019), boosting population genetic differentiation by modifying their distribution range during ENSO events. During warm phases, the distribution expands, while during cold events, it contracts. The migratory behavior of dolphinfish also reinforces this hypothesis. There are spatial changes in abundance that reflect a preference for warm temperatures (Zúñiga-Flores, Ortega-García & Klett-Traulsen, 2008; Farrell et al., 2014; Brodie, Hobday & Smith, 2015; Torrejón-Magallanes, Grados & Lau-Medrano, 2019).

The northern boundary of the TEP is a region with a seasonal convergence of three different water masses: Gulf of California, tropical surface, and transitional tropical. Additionally, the equatorial front at the TEP’s southern boundary is a convergence zone that separates the salty and cold equatorial surface water from the fresh and warm tropical surface water. Transitional zones are notably productive (Hill et al., 1998). For both transitional areas, the California and Humboldt currents are driven by winds that blow along the shore toward the equator for a substantial part of the year (Smith, 1995), generating upwelling events and driving nutrient-rich waters from deeper layers to superficial waters, promoting dense phytoplankton blooms that are the base of ocean food webs (Chan, 2019). This high productivity generates feeding grounds for many pelagic species that aggregate for various functions, including spawning (Dingle, 2014). The dolphinfish is an opportunistic feeder that consumes easily caught prey and shows seasonal changes in diet (Tripp-Valdez, Galván-Magaña & Ortega-García, 2010). The TEP boundaries are attractive for dolphinfish because of the abundance of prey species (Olson, 2001; Acha et al., 2004) and floating debris (*e.g*., driftwood, detritus, and other flotsam), with which dolphinfish are strongly associated, showing both homing and high levels of site fidelity (Girard et al., 2007; Whitney et al., 2016; Perle et al., 2020). The specific behavior in which dolphinfish tend to stay and return after foraging excursion, and their ability to orientate towards a fish aggregating device (Girard et al., 2007), support restricted gene flow within the boundaries of the study area and isolation of populations.

These seasonal genetic shifts are also supported by the barriers to gene flow identified in the region and the migration rates found in this study. The suggested differences between Chiapas with northernmost locations (Fig. 1) may be related to oceanographic conditions in the Gulf of Tehuantepec and oceanic circulation. The Gulf of Tehuantepec is influenced by intense seasonal wind events during winter, producing strong upwelling, low sea-surface temperatures, mesoscale eddies, and shallow thermocline (González-Silvera et al., 2004; Fiedler & Lavín, 2017). In addition, wind stress across Central America gives rise the poleward Costa Rica current which flows to the north reaching the Gulf of Tehuantepec where it is interrupted and restricts larval dispersal northward. Therefore, the oceanography of the Gulf of Tehuantepec may influence the genetic interchange of the Chiapas population in relation to those from northern Mexico. Although mark-recapture studies have shown that dolphinfish tend to remain residents near the areas where they were originally tagged (Kingsford & Defries, 1999; Merten, Appeldoorn & Hammond, 2014; Perle et al., 2020), some individuals have also performed longer distance migrations from northern to southern Mexico in response to seasonal temperature changes (Perle et al., 2020). This agrees with the overall low migration rates found and the asymmetric connectivity between distant regions in this study (*e.g*., from Guaymas to Chiapas), which could be facilitated by possible annual migration route during winter (Perle et al., 2020). Further, the geographic distribution of genetic variation found in this study indicated that the transoceanic connectivity between individuals in the Oceanic (OC) and Peru (PE) sites (depicted by *K* = 2 from STRUCTURE and the migration rate analysis) reflects that the deep-water zone does not constitute insurmountable barriers separating populations (Robertson & Cramer, 2009). Hence, larval dispersal promoted by oceanic currents might still eventually occur, meaning that dolphinfish populations are not entirely isolated.

Finally, the highly dynamic oceanographic processes observed in the transitional areas of the TEP may also contribute to the genetic variation observed. The mixing of water masses that differ in temperature and salinity increases the mesoscale dynamics, promoting small and long-lived energetic eddies and/or permanent upwellings (Montecino & Lange, 2009; Pantoja et al., 2012). Oceanographic conditions at transitional areas are characterized by high seasonal variations in temperature, salinity and primary productivity. North of the equator, off the cost of Mexico, the transitional area between tropical and subtropical waters known as transitional tropical water (TTW), is part of Eastern Tropical-Subtropical Convergence, where the Gulf of California water and the Tropical Surface Waters also converge (Fiedler & Talley, 2006). Strong gradients in temperature and oxygen have been reported in the TTW, originating from the thermocline and oxycline sinking in the frontal area (30 m) towards the California Current waters (90 m) (Cruz-Hernández et al., 2019). South of the equator, the equatorial cold tongue converges with the cooler water of the Peru Current, generating a front whose seasonal northern and southern limits depend on the intensity of the Peru Current (Fiedler & Lavín, 2017). The equatorial cold tongue clearly marks the limits of the tropical surface water at Central America and Mexican latitudes. This may prevent the dispersal of larvae and limit adult movements, contributing to seasonal genetic subdivisions observed in the transitional areas.

Conversely, the merging of these water masses, the strong environmental gradients, and their interaction with feeding areas and other fine-scale mechanisms of the transitional areas could promote local adaptations for individuals inhabiting them. This hypothesis agrees with previous findings of genetic differentiation in areas that exhibit environmental gradients or patchiness for other species such as *Clupea harengus* in the Baltic Sea; *Amphiprion bicinctus* in the Red Sea; and *Merluccius productus* in the Eastern Pacific (Jørgensen et al., 2005; Nanninga et al., 2014; García-De León et al., 2018). Furthermore, our AMOVA analysis reflects significant differentiation when considering the environmental differences that distinguish the biogeographic provinces. However, additional studies are required to demonstrate a link between the heterogeneous environmental conditions in these areas and the genetic differentiation found to verify that it is indeed environmental factors that drive genetic structure.

### Genetic diversity and effective population size

Historically, dolphinfish is one of the main fishery resources of many countries within the TEP, especially for Ecuador and Peru (Aires-da-Silva et al., 2016). Estimates of heterozygosity and the number of alleles in dolphinfish were in agreement with other studies of large pelagic fishes, such as *Thunnus thynnus thynnus*, *T. albacares*, *T. orientalis*, and *Xiphias gladius* (Carlsson et al., 2004; Díaz-Jaimes & Uribe-Alcocer, 2006; Nomura et al., 2014; Aguila et al., 2015; Riguik et al., 2020), as well as in a comparative study that highlighted the considerably high heterozygosity levels of marine fishes (*H*_*O*_: 0.67–0.70; Martinez, Willoughby & Christie, 2018).

Our estimates of contemporary *Ne* suggest that dolphinfish populations are below the minimum threshold of 1,000 for retaining their evolutionary potential in the long term (Frankham, Bradshaw & Brook, 2014), raising concerns that the survival of dolphinfish populations in the TEP could be in jeopardy. Deviations from the Hardy-Weinberg equilibrium were detected in some populations due to a large number of loci with heterozygote deficit, leading to positive values of inbreeding (*F*_IS_). This pattern is unlikely to be attributed to a high frequency of null alleles because loci were extensively tested for genotyping artifacts that could produce null alleles. Loci Chi389, Chi853, and Chi967 showed null allele frequencies of about 25% in only one population (one of each); however, loci had a minor effect on G_ST_ and Jost’s *D* estimates. Also, no evidence for significant allele drop-out was found using MICROCHECKER. Likewise, a Wahlund effect also seems unlikely because no cryptic genetic structure was detected using clustering analysis. Therefore, we suggest that the positive values of *F*_IS_ and the low effective population size constitute a warning that the dolphinfish populations are vulnerable to fishing pressure.

Low *Ne* estimations have been documented for other similar species such as *X. gladius* in the TEP. Estimates of *Ne* for *X. gladius* ranged from 100 (or lower) to 1,000 individuals and are comparable to those reported for *C. hippurus* in this study. The notably decreased *Ne* for *C. hippurus* parallels the remarkable decline in *X. gladius* fishery (Yüncü et al., 2020). Harvesting may increase mortality and cause deviations in drift-mutation equilibrium, which in species with short generational time may increase the loss of genetic variation over relatively few generations, which in turn is enhanced by the presence of genetic structure (Allendorf et al., 2008). Consequently, a divergent population subjected to prolonged mahi-mahi fishing practices, such as in Peru, may easily cause very low values of *Ne*. Although *C. hippurus* in Mexico is restricted to sport-recreational fishing within 50 nautical miles from the coastline (Diario Oficial de la Federación, 2013), it is common to find dolphinfish specimens within commercial landings recorded as by-catch with no official reporting, which prevents estimates of the total by-catch of this species in Mexico (Alejo-Plata, 2012). Dolphinfish show early sexual maturity, high fecundity, and an asynchronous spawning occurring in waters relatively close to the coast throughout the year in the tropics (Cheung, Lam & Pauly, 2008; Moltó et al., 2020). In the southern Gulf of California, dolphinfish reproduction occurs mainly during the warm months of summer-autumn (Zuñiga-Flores et al., 2011) whereas in southern Mexico reproduction occurs in May–July, and November–January (Alejo-Plata, Díaz-Jaimes & Salgado-Ugarte, 2011). In Costa Rica, spawning occurs in January and February (Campos et al., 1993). In Ecuador, dolphinfish spawn throughout the year, although the months of maximum recorded reproduction are October-December in the north, and from January to March in central and southern waters (Zuñiga-Flores & Lavayen, 2014). In the Economic Exclusive Zone of Peru spawning records are during austral summer (Solano et al., 2015). These studies show that *C. hippurus* spawn mainly in tropical waters, with females using coastal habitats as feeding areas more frequently than males (Alejo-Plata, 2012). As a consequence, females are more vulnerable to be capture near to the coast during the spawning season. The resulting alteration of the sex ratio would potentially reduce the effective population size and thus increase the probability of mating with relatives. Thus, it is crucial to consider that dolphinfish may be prone to genetic drift and inbreeding, which would compromise their long-term evolutionary potential.

### Mitochondrial patterns

Temporal population reductions and subsequent expansions influence genetic structures, especially for haploid and uniparental genomes. Population expansions to new areas are usually initiated by few individuals at the extremes of the distribution, followed by a rapid and large increase in the population size. We found signals of rapid population expansion of dolphinfish within the TEP, evidenced by the marked difference between haplotype (very high) and nucleotide (very low) diversities estimated for the mtDNA and the star-like phylogeny. Previous studies suggested that habitat reductions or bottlenecks are associated with low nucleotide diversity resulting from a limited number of shared sequences and numerous low-frequency haplotypes separated by few mutations (Díaz-Jaimes et al., 2006). This arrangement was found in the haplotype networks of both mitochondrial sequences and the observed pattern of nucleotide and haplotype diversity. In turn, the Bayesian Skyline plot, the unimodal mismatch distribution, and the rejection of the neutrality test clearly supported a scenario of population expansion after a period of low effective population size in our samples of dolphinfish. One possible explanation is that fragments of populations of dolphinfish when faced with unfavorable environmental conditions may survive in refugia. Upon restoration of favorable environmental conditions, these populations could rapidly rebound, colonize new sites and continue to grow over subsequent generations. The timing of expansion as estimated from the Bayesian Skyline plot was about 126,000 years before present for ND1 and 92,838 for CYTB; these periods coincide with the penultimate interglacial period during the late Pleistocene. This period was characterized by sea temperature two to three degrees warmer than the current values and a sea level five to six meters higher than present (reviewed by Bergoeing, 2017). This is not the first study that associates mitochondrial genetic variation in *C. hippurus* with population expansion after cooling episodes during the Pleistocene, and cycles of population contraction and expansion following changes in sea surface temperature and habitat availability in the Eastern Pacific (Díaz-Jaimes et al., 2006).

It has been previously hypothesized that a lack of genetic divergence among *C. hippurus* populations could be due to extensive region-wide gene flow (Díaz-Jaimes et al., 2006; Tripp-Valdez et al., 2010) or because genetic drift could not impact genetic variation due to large population size (Díaz-Jaimes et al., 2010). Even if oceanographic features such as eddies, gyres, and fronts with substantial environmental variability can limit the dispersal of larvae or more mobile life stages, a large effective population size counteracts the effects of genetic drift on genetic variation, blurring evidence of population subdivision (Díaz-Jaimes et al., 2010). Thus, we propose that the low genetic differentiation in microsatellite markers of *C. hippurus* indicates a recent genetic divergence with enhanced genetic drift in populations at the latitudinal extremes of the species range, rather than the previous postulation of high gene flow among all populations.

## Conclusions

Although the marine environment is assumed to be an open ecosystem, genetic differentiation is more frequent than previously thought because environmental variability and oceanographic systems can influence gene flow among populations through direct dispersal or by currents. We found a clear pattern of genetic structure at the latitudinal limits of the studied species distribution and hypothesize that cycles of expansion-contraction following temperature changes are responsible for seasonal population subdivision in *C. hippurus*, promoting differentiation. However, we cannot rule out the possibility that oceanographic dynamics also contribute to the observed pattern. Although this marine species is highly abundant and exhibits high genetic diversity, the relatively low *Ne* values could jeopardize long-term survival of *C. hippurus*, which must be considered when establishing regional management plans. Finally, temporal genetic variability needs to be assessed in future studies in order to verify whether allele frequencies change over time in response to harvesting.

## Supplemental Information

10.7717/peerj.14389/supp-1Supplemental Information 1Evaluation of the *K* values (genetic clusters) using different approaches and *K* = 2 and *K* = 3 from TESS3.(A) Cross-validation score from TESS 3 in which smaller values mean better runs. (B) The proportion of estimated ancestry with *K* = 2 and *K* = 3 from TESS 3 using 311 individuals of *Coryphaena hippurus* from the Tropical Eastern Pacific. Each individual is represented by a vertical line with the assignment probability to each of the clusters (*K*) proportional to the length of each color. Population names are in Table 1. (C) Values of Bayesian Information Criteria (BIC) in which the optimal clustering solution is indicated by an elbow in the curve (*i.e*., the lowest BIC). (D) The uppermost hierarchical level of the genetic partition by $\Delta K$ values [orange line in (D)] and the mean posterior probability (Ln P(D)) for each *K* [black line in (D)].

10.7717/peerj.14389/supp-2 Supplemental Information 2 Probabilities of assignment analysis for mitochondrial DNA data. Plots from Bayesian assignment analysis for *K* = 2 of *Coryphaena hippurus* based on a fragment of the mitochondrial gene NADH subunit 1 (ND1; upper) and Cytochrome B (CYTB; lower) in the Tropical Eastern Pacific. Population names are in Table 1.

10.7717/peerj.14389/supp-3 Supplemental Information 3 Barriers to gene flow for microsatellite data of *Coryphaena hippurus* within the Tropical Eastern Pacific. Yellow lines represent the three main geographic barriers indicated with letters a, b, and c. Voronoi tessellation is shown in green, and in color blue the corresponding Delaunay triangulation of samples (dots and numbers in red). Black numbers indicate bootstrap support. Populations label code: 1 = Bahía Magdalena (BM); 2 = Punta Lobos (PL); 3 = Cabo San Lucas (CSL); 4= Guaymas (GY); 5 = Mazatlán (MAZ); 6 = Chiapas (CH); 7 = Oceanic sample (OC); 8 = Ecuador (EC); 9 = Peru (PE).

10.7717/peerj.14389/supp-4 Supplemental Information 4 Null allele frequencies based on Oosterhout method.

10.7717/peerj.14389/supp-5 Supplemental Information 5 Test of Hardy Weinberg Equilibrium by locus. First probability test and then, when H1 = heterozygote deficit

10.7717/peerj.14389/supp-6 Supplemental Information 6 Linkage disequilibrium probabilities. Linkage disequilibrium between pairs of loci.

## Additional Information and Declarations

### DNA Deposition

The following information was supplied regarding the deposition of DNA sequences: The mtDNA sequences are available at GenBank: MZ725384–MZ725448. The genotypes for microsatellites are available at Zenodo: Ochoa-Zavala, Maried, Díaz-Jaimes, Píndaro, Ortega-García, Sofía, & Galván-Magaña, Felipe. (2022). Nuclear genotypes from *Coryphaena hippurus* [Data set]. Zenodo. https://doi.org/10.5281/zenodo.6949509.

### Data Availability

The following information was supplied regarding data availability: The genotypes from the 14 nuclear microsatellites of *C. hippurus* are available at Zenodo: Ochoa-Zavala, Maried, Díaz-Jaimes, Píndaro, Ortega-García, Sofía, & Galván-Magaña, Felipe. (2022). Nuclear genotypes from *Coryphaena hippurus* [Data set]. Zenodo. https://doi.org/10.5281/zenodo.6949509.
