# Supplementary figures and images for: Genetic divergence at species boundaries of the dolphinfish (Coryphaena hippurus) in the Tropical Eastern Pacific
Source: PeerJ. 2022 Nov 17;10:e14389. doi: 10.7717/peerj.14389 (PMC9676019; doi:10.7717/peerj.14389)

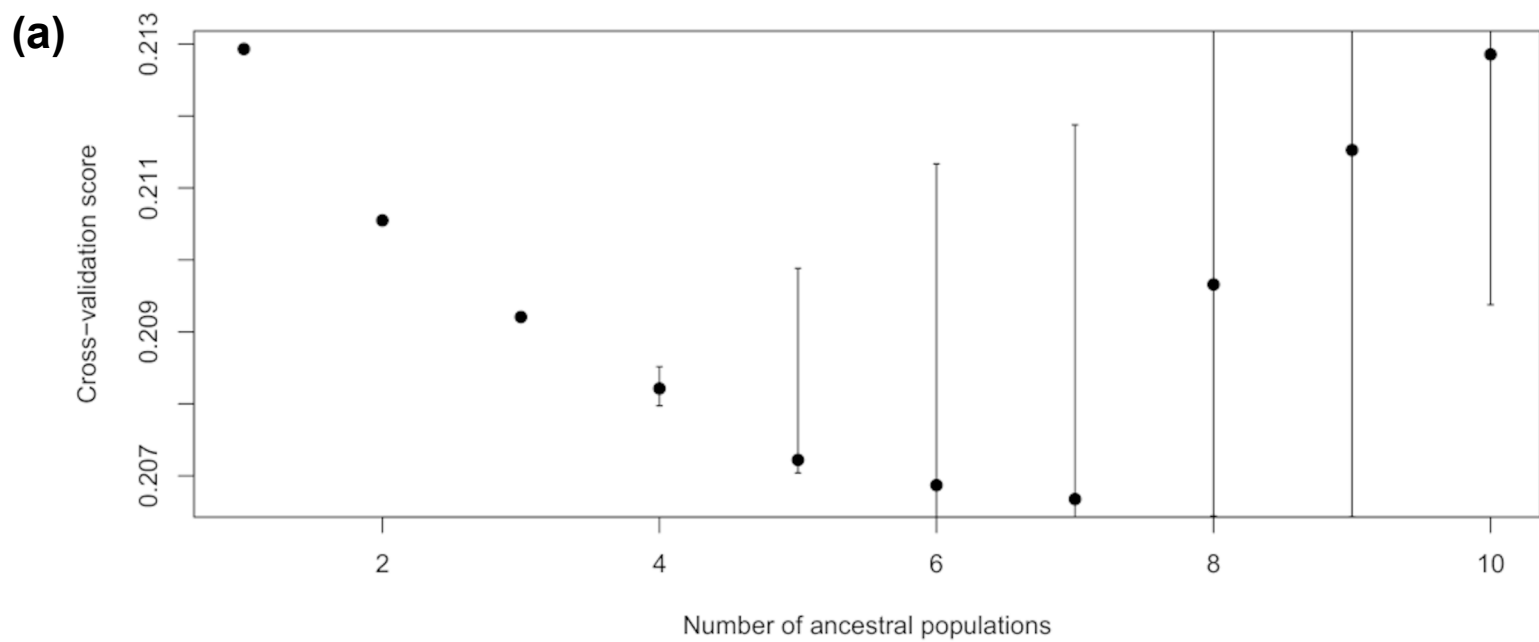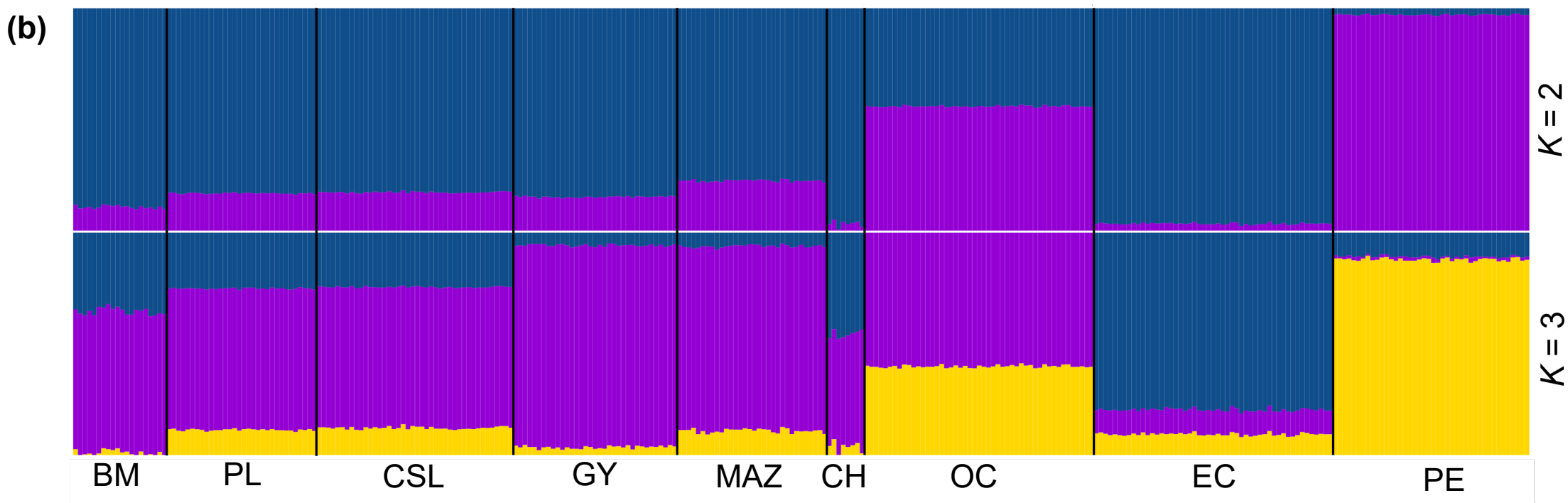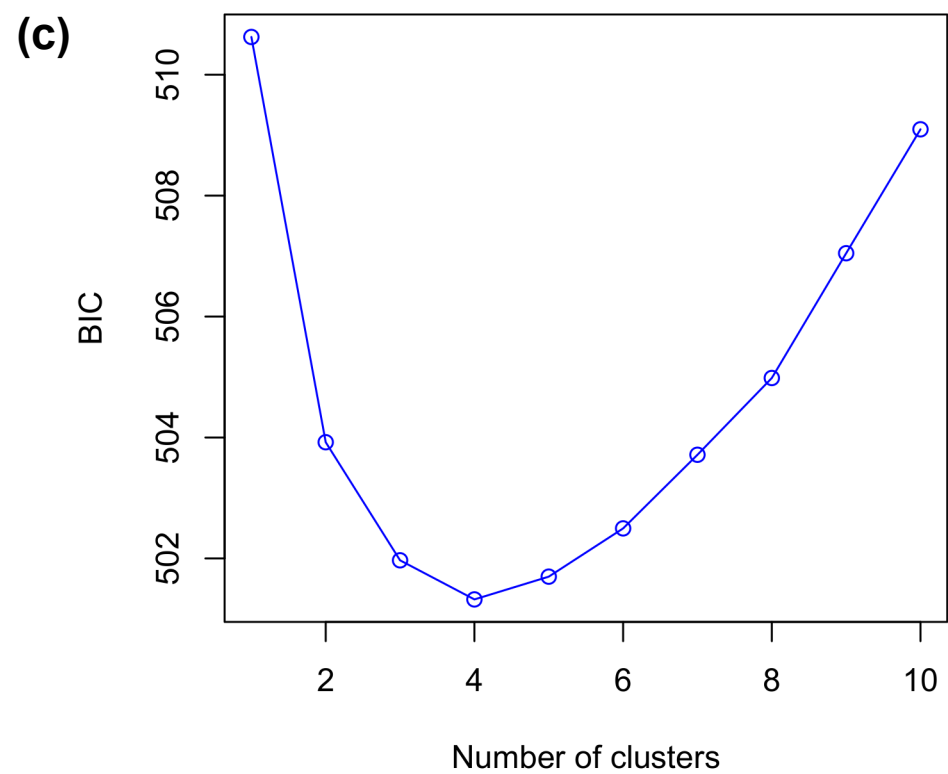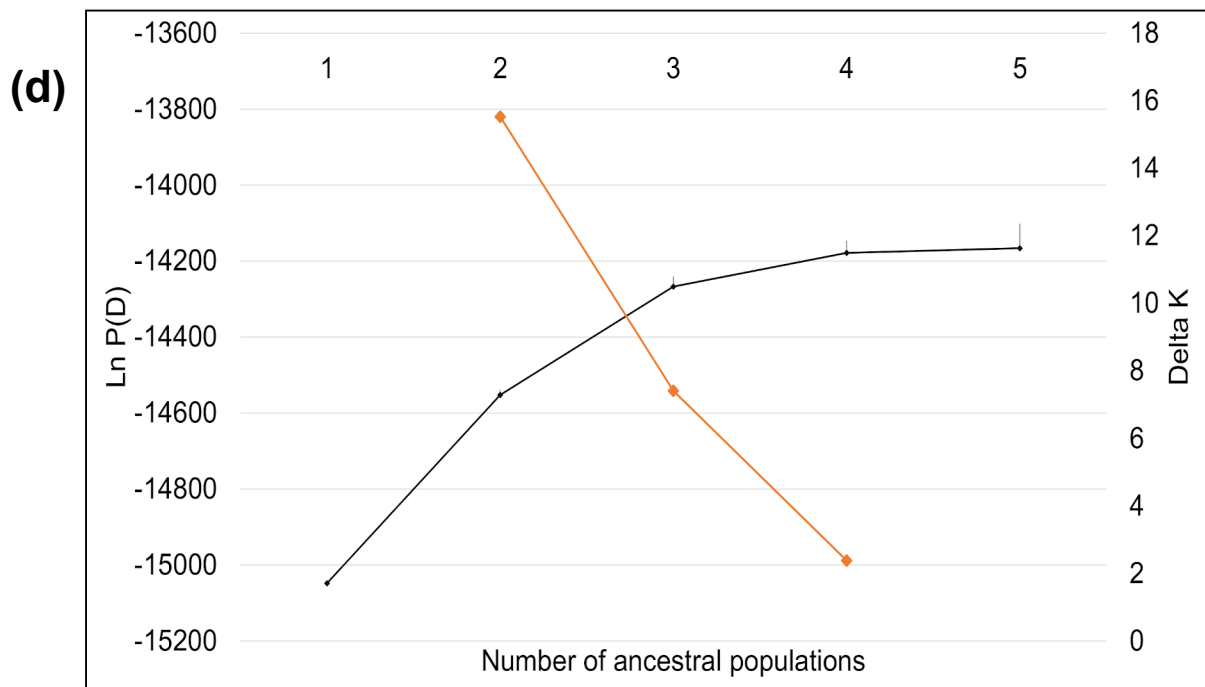

Supplement: Supplemental Information 1 — (A) Cross-validation score from TESS 3 in which smaller values mean better runs. (B) The proportion of estimated ancestry with K = 2 and K = 3 from TESS 3 using 311 individuals of Coryphaena hippurus from the Tropical Eastern Pacific. Each individual is represented by a vertical line with the assignment probability to each of the clusters (K) proportional to the length of each color. Population names are in Table 1. (C) Values of Bayesian Information Criteria (BIC) in which the optimal clustering solution is indicated by an elbow in the curve (i.e., the lowest BIC). (D) The uppermost hierarchical level of the genetic partition by $\Delta K$ΔK values [orange line in (D)] and the mean posterior probability (Ln P(D)) for each K [black line in (D)]. [file peerj-10-14389-s001.pdf]

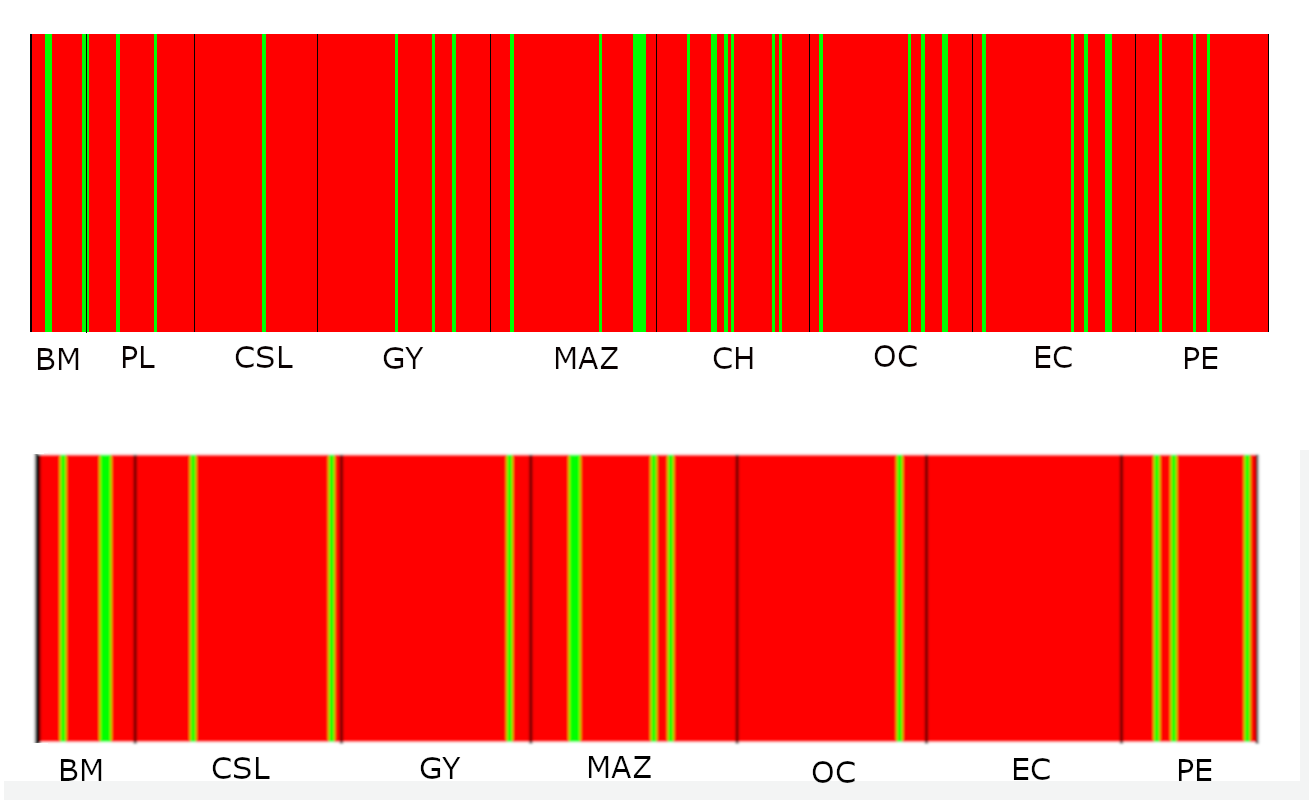

Supplement: Supplemental Information 2 — Plots from Bayesian assignment analysis for K = 2 of Coryphaena hippurus based on a fragment of the mitochondrial gene NADH subunit 1 (ND1; upper) and Cytochrome B (CYTB; lower) in the Tropical Eastern Pacific. Population names are in Table 1. [file peerj-10-14389-s002.png]

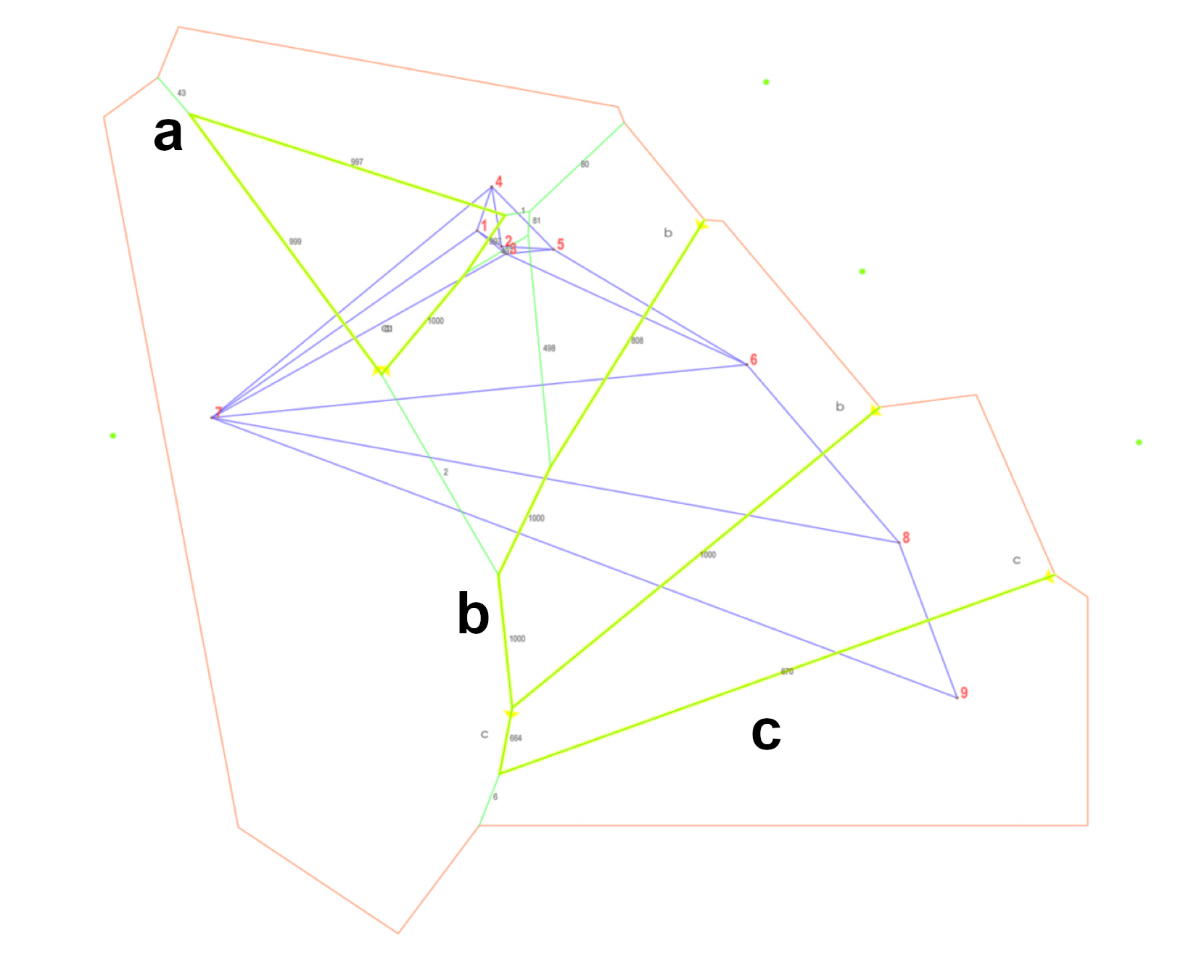

Supplement: Supplemental Information 3 — Yellow lines represent the three main geographic barriers indicated with letters a, b, and c. Voronoi tessellation is shown in green, and in color blue the corresponding Delaunay triangulation of samples (dots and numbers in red). Black numbers indicate bootstrap support. Populations label code: 1 = Bahía Magdalena (BM); 2 = Punta Lobos (PL); 3 = Cabo San Lucas (CSL); 4= Guaymas (GY); 5 = Mazatlán (MAZ); 6 = Chiapas (CH); 7 = Oceanic sample (OC); 8 = Ecuador (EC); 9 = Peru (PE). [file peerj-10-14389-s003.jpg]
